# Assessment of a Hands-On Seminar on Gastrointestinal Ultrasound

**DOI:** 10.3390/healthcare8040541

**Published:** 2020-12-07

**Authors:** Masaaki Yamada, Yuichi Hasegawa, Seiji Yamashiro, Michikazu Sekine, Yukihiro Asano, Haruka Fujinami

**Affiliations:** 1 Department of Epidemiology and Health Policy, School of Medicine, University of Toyama, 2630 Sugitani, Toyama 930-0194, Japan; sekine@med.u-toyama.ac.jp; 2 Department of Clinical Laboratory, Narita Red Cross Hospital, Iida 90-1, Narita, Chiba 286-0041, Japan; hasegawa@choshinet.or.jp (Y.H.); y-asano@wb3.so-net.ne.jp (Y.A.); 3 Department of General Medicine, Toyama University Hospital, 2630 Sugitani, Toyama 930-0194, Japan; yamashir@med.u-toyama.ac.jp; 4 Department of Gastroenterology and Hepatology, School of Medicine, University of Toyama, 2630 Sugitani, Toyama 930-0194, Japan; haruka52@med.u-toyama.ac.jp

**Keywords:** bowel, ultrasonography, systematic scan, transabdominal

## Abstract

Transabdominal gastrointestinal (GI) ultrasound (US), despite its utility, is not a common procedure and underappreciated owing to its difficulty to perform. This study aimed to disseminate the skills of GIUS and assess the impact of our hands-on seminar. We annually held a half-day, hands-on seminar on GIUS at University of Toyama Hospital for physicians and sonographers from 2015 to 2017. Two months after the seminar, we inquired about clinical attainment by questionnaire. Out of 55 participants, 46 (83.6%) returned their questionnaires. Twenty participants (43.5%) reported that they had successfully diagnosed at least one GI disorder via GIUS since the seminar. Residual analyses stratified by the participants’ background showed that the novices, those having < 2 years’ experience in performing abdominal US, or no prior knowledge of GIUS, had significantly lower attainment rates (23.5% and 12.5%, respectively) than the others. Participants with 2 to 5 years’ experience in performing abdominal US or with some knowledge of GIUS had much higher rates of diagnosing GI disorders (54.5% and 57.9%, respectively). Nearly half of the participants had identified GI disorders using GIUS in 2 months following the training. The hands-on seminar was beneficial in disseminating these skills among a wide range of US operators.

## 1. Introduction

Numerous different specialties use ultrasound (US) examinations in a focused manner, such as the point-of-care ultrasound (POCUS) examination [1,2], and this technique is gaining popularity among specialists and general practitioners worldwide. However, the clinical value of transabdominal ultrasound (US) of the gastrointestinal (GI) tract remains under-appreciated [3]. This is because the GI tract is often not visualized on transabdominal US due to bowel gas and stool [3,4], thus most US operators are reluctant to perform GIUS. Abdominal symptoms are one of the most common causes for patients visiting clinics and emergency departments. According to statistics on emergency calls, abdominal pain was the second most frequent cause of ambulance calls in Tokyo in 2015, following children’s head trauma [5], and GI disorders, such as intestinal infection, acute appendicitis, and bowel obstruction are common etiologies among inpatients with acute abdominal pain in Japan [6]. Thus, GIUS it is beneficial to all physicians, emergency doctors, and sonographers who see patients with GI disorder.

One of the most significant challenges for the widespread dissemination of GIUS is its operator dependency. Although practice guidelines for acute abdominal pain recommend US as a beneficial diagnostic tool for patients with non-GI tract disorders such as cholecystitis and hydronephrosis, and GI disorders such as appendicitis, as well as diverticulitis, especially for women and children, it simultaneously warns that the accuracy of diagnosis by US varies with sonographer’s expertise [7,8]. Consequently, many doctors rely on endoscopy or computed tomography (CT) despite the invasiveness or radiation exposure, believing that abdominal US is not useful in evaluating the GI tract [4]. In addition to physicians in Japan, medical technologists conduct medical examinations such as blood tests, laboratory culture, and US under supervision. Those medical technologists who mainly perform US examinations are called sonographers [9]. A questionnaire survey among sonographers also showed that the percentage of GI-tract screening done was only 39.8% (97/244) [10]. Most of them did not screen GI tracts on routine abdominal US because of the difficulty in performing GIUS.

Nevertheless, GIUS has been useful in diagnosing GI tract disorders, such as bowel obstruction, appendicitis, diverticulitis, intussusception, and inflammatory bowel diseases [3,4,11,12,13]. However, the number of operators conducting GIUS in these studies was notably limited [11,13]. Thus, a practical seminar or training is needed to increase the number of GIUS operators and to develop the skill among physicians and sonographers.

Thus far, didactic and hands-on seminars on GIUS have taken place around Japan [14,15]. However, to the best of our knowledge, the effects of these seminars have not been reported. For instructors or staff holding these seminars and for potential GIUS operators, information about the effectiveness of GIUS seminars can be useful. We hypothesized that a hands-on seminar on GIUS, teaching not only theoretical knowledge but also motor skills (e.g., handling transducer and manipulating machine), could help US operators to perform systematic scanning. We expect that learning GIUS gives them a sense of purpose in screening GI tracts and helps them gradually improve their skills in clinical settings. The purpose of our study was to assess the impact of the hands-on seminar on performing GIUS in clinical settings.

## 2. Methods and Participants 

### 2.1. Study Design 

This was an observational prospective cohort study, assessing the effects of the GIUS hands-on seminar on the participants. Our studies were performed following the instruction from a member of the Ethics Committee of the University of Toyama. The official approval from the Ethics Committee was exempted because we collected only characteristics and educational appraisal from participants. We explained the purpose of the questionnaires to the participants and only those who provided written consent were included in our analyses. Our study was in accordance with the Helsinki Declaration of 1964 and later versions.

### 2.2. Gastrointestinal Ultrasound Seminar

**Figure 1:**
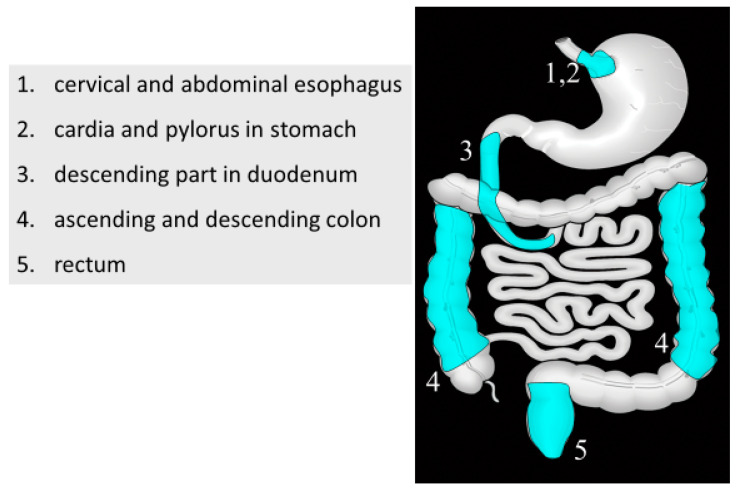
Fixed part in each bowel organ in systematic scanning. The cervical and abdominal esophagus in esophagus, parts of cardia and pylorus in stomach, descending part in duodenum, ascending and descending colon in large intestine, and rectum.

**Figure 2:**
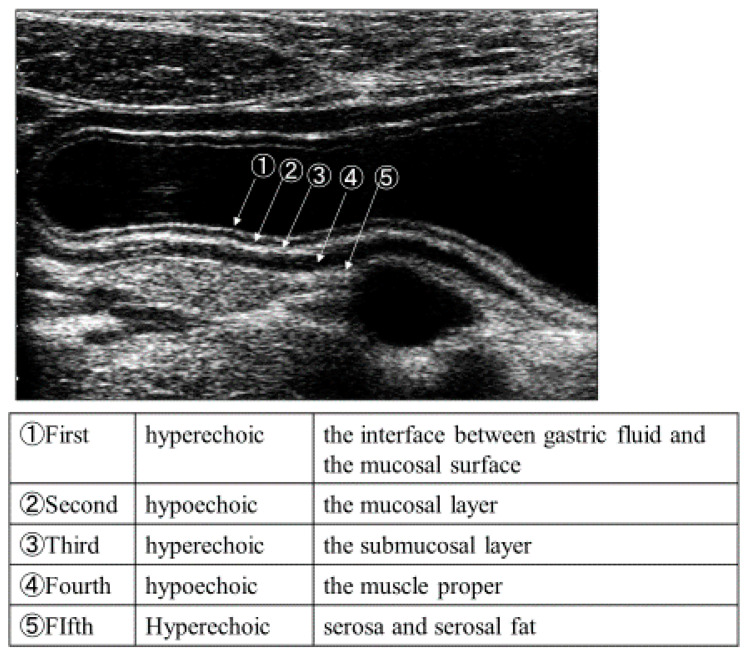
Five-layer stratification of stomach by sonography. The first inner hyperechoic layer represents the interface between gastric fluid and the mucosal surface; the second hypoechoic layer shows the mucosal layer; the third hyperechoic layer shows the submucosal layer; the fourth hypoechoic layer shows the muscle proper; and the fifth hyperechoic layer shows serosa and serosal fat.

From 2015 to 2017, we annually held a half-day, hands-on seminar on GIUS at University of Toyama Hospital. In performing GIUS, systematic scanning is recommended to allow visualization of the entire GI tract [16,17]. Operators first need to detect the fixed parts in each GI tract. Thereafter, they can identify the anatomical structures and carefully analyze images of the GI tract (Figure 1 and Figure 2). However, this first procedure, the detection of fixed parts, is a major challenge to performing GIUS if the US operator is not familiar with images of bowel gas and stool. Properly understanding systematic scanning is imperative to effectively perform GIUS.

Table 1 shows an overview of the seminar. The seminar consisted of three sessions: upper GI tract (esophagus, stomach, and duodenum), lower GI tract (small and large intestine), and the appendix. Each session lasted for about 60 min. Introductory lectures, which covered the basic anatomy and five-layer stratification of the GI tract and the systematic scanning procedure, were included in each session. In addition, still and animated images of pathological lesions were illustrated to teach the ten factors for diagnosing disorders; (1) wall thickness, (2) site and distribution of the lesion, (3) wall stratification of the five layers, (4) echogenicity of the lesion, (6) peristalsis, (7) compliance and compressibility, (8) luminal stenosis or dilatation, (9) deformity, and (10) blood flow [16]. Following the lecture, the participants were divided into three groups. In each group, each participant received a face-to-face hands-on training for six minutes. All the instructors in the seminars were certified as “Registered Senior Medical Sonographers (RSMS)” by the Japanese Society of Ultrasonics in Medicine [18].

### 2.3. Seminar Participants, Subjects, Machines and Questionnaire Survey

Physicians or sonographers with a wide range of experience in performing abdominal US were recruited for the GIUS hands-on seminar. The participants were from several hospitals in the Toyama Prefecture, Japan, and volunteered to register in advance because the number of instructors (RSMS), volunteer subjects, and US machines were limited. Healthy medical students of the university where

We performed volunteered as subjects. US machines, Aplio 500 and Aplio 400 (Toshiba Medical Systems, Tokyo, Japan (now Canon Medical Systems)), were used in the seminar.

**Figure 3:**
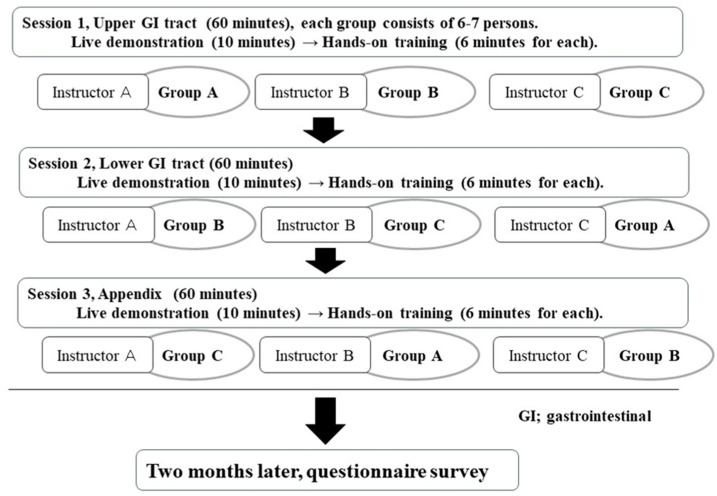
Flow chart of hands-on seminar and follow-up survey.

Before the seminar, we asked the participants about their characteristics: occupation, years of experience in performing abdominal US, and knowledge of GIUS. The knowledge-related question was “How much do you know about GIUS (US for esophagus, stomach, duodenum, small and large intestine, and appendix)?” Respondents answered the question using a 4-point scale (1, no; 2, almost no or a little; 3, some extent; 4, yes, fully). Two months after the hands-on seminar, we sent a questionnaire to the participants to examine their clinical attainment of GIUS (Figure 3). The questions asked were “Have you ever detected any GI disorders by GIUS since the hands-on seminar?” and “If yes, what kind of disorder did you diagnose?”

### 2.4. Statistical Analysis

Participant’s clinical attainment, which we see as an effect of the hands-on seminar after two months, were compared according to the participants’ occupation, experience in performing abdominal US (years), and preliminary knowledge of GIUS, using the chi-square test, trend test, and residual analyses. All analyses were performed using SPSS version 25.0 J (SPSS, Chicago, IL, USA). A two-tailed *p*-value of less than 0.05 was considered to be statistically significant.

## 3. Results

Out of 55 participants, 46 returned their questionnaires (response rate 83.6%). Twenty-eight physicians (5 junior residents, 8 gastroenterologists, and 15 general physicians) and 18 sonographers were included in our analyses (Table 2). More than half (28/46) had less than five years’ experience in performing abdominal US. Regarding knowledge of GIUS prior to the hands-on seminar, about three-fourth participants answered “no” or “a little.”

Table 3 shows the effect of the GIUS hands-on seminar on participants two months later. Generally, 43.5% of participants (20/46) had at least one experience of detecting a GI disorder. Analyses stratified by the participant’s characteristics showed that participants with more experience in performing abdominal US or more knowledge of GIUS were more likely to have diagnosed GI disorders (trend test, *p* < 0.05 and *p* < 0.01, respectively). Residual analyses showed that only the novice participants, with <2 years’ experience in performing abdominal US or no prior knowledge on GIUS, had significantly lower attainment rates (23.5% and 12.5%, respectively). Participants with 2 to <5 years’ experience in performing abdominal US or with a little knowledge of GIUS had much higher rates of diagnosing any GI disorders in the two months after the seminar (54.5% and 57.9%, respectively) than the novice participants.

Table 4 shows the disorders diagnosed by the participants as follows: bowel obstruction, infectious and ischemic colitis, and malignant lesions. Some of them identified two or more GI disorders.

## 4. Discussion

Despite the fact that most participants did not have prior knowledge of GIUS, our survey demonstrated that nearly half of the participants had successfully detected GI lesions and that, except for the novice participants, more than half of them successfully diagnosed GI disorders via GIUS. The hands-on GIUS seminar was beneficial in developing the skill of performing GIUS among a wide range of US operators.

Several studies have assessed the effects of US seminars in POCUS [19,20,21], FAST (focused assessments with sonography for trauma) [22], breast US [23], and transesophageal echocardiography [24]. To the best of our knowledge, this is the first study assessing the effects of a hands-on seminar on GIUS. We previously reported on a comprehensive test administered to participants after they joined the didactic GIUS seminar, which did not include hands-on training [25]. The findings showed that only the experts in abdominal US satisfactorily comprehended the still and animated images of the GI tract. Attendees with less than five years’ experience in performing abdominal US did not comprehend the images of the GI tract. We hypothesized that a hands-on seminar on GIUS could have more positive effects than the didactic seminar because the hands-on seminar would reinforce the anatomical knowledge of the GI tract and motor skills (transducer handling and machine manipulation). Knudsen et al., in a randomized trial of hands-on versus no hands-on US training in anatomy among medical students, reported that faster interpretation of US images and higher motivation scores were noted in the hands-on group [26]. Because we did not include a didactic group in this study, we could not compare the effect between didactic and hands-on training. However, a hands-on GIUS seminar was considered beneficial to participants with a wider range of experience levels compared to the didactic seminar.

Of note, novice participants, showed significantly lower attainment rates in identifying GI disorders. A possible explanation is that they could not identify the fixed parts in each organ, such as the cardia and antrum in the stomach and the ascending and descending colon in the large intestine. These parts are key to identifying the anatomical structures in the systematic scanning of the GI tract. Without identifying the fixed parts, US operators can neither focus on the images of the GI tract nor use them successfully in diagnosis based on the ten outlined factors. Basic skills in performing abdominal US were considered to be a minimum requirement to learn GIUS.

GI disorders commonly identified in this study included bowel obstruction, infectious colitis, appendicitis, ischemic colitis, and gastric cancer. For participants who missed GI diagnosis, we recommend learning by comparing images of GIUS to those of other modalities like CT, and to try to identify ischemic colitis and infectious colitis. This is because these disorders are relatively detectable even for novice trainees. Unfortunately, trainers who can teach GIUS rarely exist in most hospitals. However, it is quite educational for all participants to ascertain (or compare) the images of GIUS on their own, even after GI disorders were diagnosed by CT or Endoscopy (or Colonoscopy).

Several participants reported that they had successfully diagnosed two or more GI disorders, and notably, there were a few novice participants among them. In the seminar there were several doctors and sonographers who worked at the same hospital with the novice participants. We considered that the novices benefited from the cluster effect, as they were surrounded by colleagues or senior physicians with enthusiasm for GIUS. Rather than learning GIUS alone, learning with colleagues in the same hospital may generate a more educational environment.

### Study Strengths and Limitations

Regarding the strengths of this study, we examined the beneficial effects of conducting a hands-on seminar on GIUS, and we are not aware of any other report of GIUS. In addition, we collected a wide range of participants with regard to occupation and experiences in performing abdominal US, thus enhancing the generalizability of our results. Our results can, therefore, serve as a yardstick for instructors or staff holding GIUS hands-on seminars, to compare their effects in the future. Despite these strengths, our study has some limitations.

First, our study size was limited by the lack of instructors, US machines, volunteer subjects, and seminar staff for the hands-on seminar. Although we included participants with a wide variety of US skills, those with a strong motivation to learn GIUS might have opted for this seminar. A future study with a larger number of participants should be conducted to increase the validity and examine the cluster effect. Second, clinical attainments were based on the participants’ answers; therefore, our study is subject to misdiagnosis such as detecting a lesion which was, actually, the contracted intestine, or diagnosing swollen lymph nodes as appendicitis. When learning the skill of GIUS in clinical settings, all participants are required to compare their US images to that of CT or Endoscopy (or Colonoscopy). Despite the lack of a validated answer in this study, we consider that our hands-on seminar raised participants’ awareness of GIUS and encouraged them to perform GIUS after the seminar. In the future, a study providing specificity, sensitivity and predictive value by collecting images of GIUS and other modalities should be conducted. Third, despite an objective measure, only the years of abdominal US experience were used to judge participants’ US skill prior to the seminar. The total number of clinical cases in performing abdominal US and in other US modalities, such as point-of-care US and obstetric US, and any other confounders should be clarified in future studies.

## 5. Conclusions

Although GIUS is not commonly performed due to its procedural difficulty, we aimed to disseminate GIUS-related skills by conducting a hands-on seminar and assessing the effects on participants. Our data revealed that US operators with more experience in performing abdominal US or more knowledge of GIUS were more likely to have diagnosed GI disorders. Furthermore, nearly half of seminar participants successfully diagnosed at least one GI disorder via GIUS in the two months following the training. This study demonstrates that hands-on seminars are beneficial to performing GIUS among a wide range of US operators.

## Figures and Tables

**Table 1:** Overview of the hands-on seminar.

Sessions	Contents
Introductory lecture and live demonstration (in each session for about 10 min)	Anatomy and histology of the GI tract, and how to perform the systematic scanning (including ways to identify the fixed part in each part)
Session 1, Upper GI tract	Esophagus: fixed part (cervical and abdominal esophagus)
(Total 50 min, 6 min for each participant)	The cervical portion behind the thyroid gland and the abdominal portion behind the left lobe of the liver.
	Stomach: fixed part (cardia and pylorus).
	The esophagogastric junction, cardia, body behind the liver, and antrum in front of the pancreas.
	Duodenum: fixed part (descending part)
	Bulb near the gallbladder, the descending part along the head of the pancreas, and the horizontal part between the aorta and SMA
	Small intestine: no fixed part
	The jejunum in the upper quadrants and the ileum in the lower quadrants, which show stronger peristalses compared to the large intestine.
Session 2, Lower GI tract	Large intestine: fixed part (ascending and descending colon and rectum)
(Total 50 min, 6 min for each participant)	Identifying the ascending and descending colon, then scanning the transverse colon between them, the rectum behind the prostate, or the uterus.
Session 3, Appendix (Total 50 min, 6 min for each participant)	Appendix is not fixed. Identifying the Bauhin valve in the cecum, then slightly moving the probe to the lower side, and finally identifying the beak sign of the appendix.

GI; gastrointestinal, SMA; superior mesenteric artery.

**Table 2:** Characteristics of the hands-on seminar participants (*n* = 46).

Background		*N*	%
Type of Occupation	Physicians	28	60.9
	Junior resident	5	10.9
	Gastroenterologist	8	17.4
	General physician	15	32.6
	Sonographer	18	39.1
Experience in performing abdominal US (Years)	0 to <2	17	37.0
	2 to <5	11	23.9
	5 to <10	8	17.4
	10 or more	10	21.7
Knowledge of GIUS (prior to hands-on seminar)	No	16	34.8
	almost no or a little	19	41.3
	some extent	9	19.6
	yes (fully)	2	4.3

US, ultrasound; GIUS, gastrointestinal ultrasound.

**Table 3:** Identifying any GI disorder two months after the hands-on seminar (*n* = 46).

Background		*N*	%	Chi-Square	Trend Test	Residual (Adjusted)
Type of Occupation	Physicians	12/28	42.9	0.91	N.A	−0.1 0.1
	Sonographer	8/18	44.4			
Experience in performing abdominal US (Years)	0 to <2	4/17	23.5	**	*	−2.1 * 0.8 −0.4 1.9
	2 to <5	6/11	54.5			
	5 to <10	3/8	37.5			
	10 or more	7/10	70.0			
Knowledge of GIUS (prior to hands-on seminar)	no	2/16	12.5	**	**	−3.1 ** 1.7 1.5
	almost no or a little	11/19	57.9			
	some extent or more	7/11	63.6			

GI, gastrointestinal; US, ultrasound; GIUS, gastrointestinal ultrasound; N.A, not applicable. *p*-value: * *p* < 0.05, ** *p* < 0.01.

**Table 4:** GI disorders diagnosed by the hands-on seminar participants (*n* = 20).

GI Disorder	Number
Bowel obstruction	4
Infectious colitis	4
Appendicitis	4
Ischemic colitis	3
Gastric cancer	3
Others (lymphoma, constipation, and so forth)	2

GI, gastrointestinal. (Some participants diagnosed two or three GI disorders).
